# Normalization of two-channel microarrays accounting for experimental design and intensity-dependent relationships

**DOI:** 10.1186/gb-2007-8-3-r44

**Published:** 2007-03-28

**Authors:** Alan R Dabney, John D Storey

**Affiliations:** 1 Department of Statistics, Texas A&M University, College Station, TX 77843, USA; 2 Department of Biostatistics and Department of Genome Sciences, University of Washington, WA 98195, USA

## Abstract

In normalizing two-channel expression arrays, the ANOVA approach explicitly incorporates the experimental design in its model, and the MA plot-based approach accounts for intensity-dependent biases. However, both approaches can lead to inaccurate normalization in fairly common scenarios. We propose a method called efficient Common Array Dye Swap (eCADS) for normalizing two-channel microarrays that accounts for both experimental design and intensity-dependent biases. Under reasonable experimental designs, eCADS preserves differential expression relationships and requires only a single array per sample pair.

## Abstract 2

eCADS is a new method for multiple array normalization of two-channel microarrays that takes into account general experimental designs and intensity-dependent relationships and allows for a more efficient dye-swap design that requires only one array per sample pair.

## Background

The two-channel microarray continues to be an important platform for characterizing genomewide expression levels. For example, a two-channel array technology using inkjet printing techniques was recently introduced by Agilent Laboratories (Palo Alto, California) that combines some of the favorable properties of single-channel oligonucleotide arrays and two-channel cDNA arrays. A recent paper compared one-channel and two-channel platforms, and concluded that the two approaches are basically equivalent in terms of reproducibility, sensitivity, and specificity [1]. In comparison with the single-channel platform, then, the two-channel platform basically doubles the number of comparisons that can be made between two groups using a fixed number of arrays, when the efficient dye-swap design proposed here is employed.

As with all high throughput array-based technologies, it is necessary to preprocess two-channel gene expression arrays to account for systematic biases [2-7]. In particular, there is evidence of dye bias, or systematic differences between the incorporation rates of the fluorescent dyes used for labeling targets. There may also be systematic differences between expression measurements on the same sample but different arrays, representing array effects. Other sources of bias include spatial trends on arrays and so-called batch effects.

In order to make reliable conclusions based on these data, it is necessary to take into account all sources of signal, both biological and systematic. Early work carried out preprocessing and statistical inference simultaneously [8]. However, it has become common practice to carry out 'normalization' as a preprocessing step, adjusting the raw expression profiles so that all systematic biases have been removed and carrying out all subsequent analyses without consideration for the preprocessing [9,10].

A standard normalization method involves smoothing so-called MA plots [2-4]. An MA plot compares differential expression to overall intensity. MA methods such as lowess smoothing of MA plots remove any observed relationship between differential expression and overall intensity. An important property of this approach is that each array is adjusted separately and the experimental design is not taken into account when doing so. It has been shown that MA methods make strong assumptions that are difficult to validate in practice [11]. When these assumptions are violated, MA methods introduce errors into subsequent inference. In particular, MA methods can introduce large-scale spurious differential expression while at the same time removing true differential expression signal [11].

Alternative normalization methods can be derived from analysis of variance (ANOVA) models [8,9], with dye-swap averaging a simple example. However, it is difficult to incorporate complex biases (the intensity-dependent biases targeted by MA methods, for example) using classic ANOVA models. The classic ANOVA model's use of factor terms to parameterize biases can lead to underfitting of some bias sources and overfitting of others.

**Figure 1:**
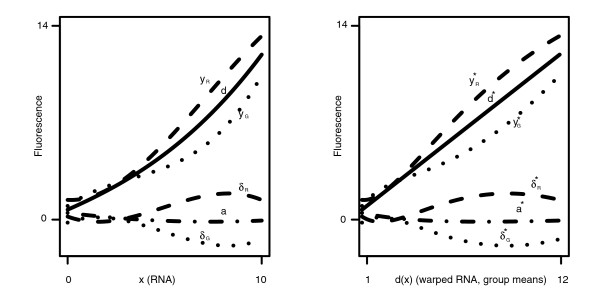
Overview of the eCADS model. The left panel summarizes the model of equation 2. The observed fluorescence intensity (*y*_*R *_or *y*_*G*_) for agene with *x *RNA is modeled as the sum of the average dye function *d*, the function corresponding to the dye used for labeling (*δ*_*R *_or *δ*_*G*_), and an array-specific function *a*. Since we do not know the true RNA amounts *x*, we warp the *x*-axis so that every *x *value is replaced with *d*(*x*); these 'warped RNA amounts' are essentially group means adjusted gene-by-gene for bias (see Model formulation). The curves in the right panel are analogously warped versions of the curves in the left panel, now representing deviations from the group mean (the straight line). The warping enables the estimation of the model without affecting the relationships of interest.

With these issues in mind, we developed a general intensity-dependent model (see equation 2) for the normalization of two-channel microarrays. The model assumes that, in the absence of bias, observed log fluorescence intensity is a monotone function of true 'RNA amount', a convenient abstraction. Intensity-dependent biases result from functions of RNA amount. Equation 2 includes functions for dye and array biases, but can include additional terms as warranted by the experimental design; namely, one would want to include any variables that may have a widespread effect on expression. An illustration of the model is given in the left panel of Figure 1. Normalization is carried out by subtracting off the terms in the model that represent bias.

While it seems natural to model intensity-dependent relationships as functions of RNA amount, there is a challenge in that we can not directly estimate RNA amount. Instead, we estimate a particular monotone function of RNA amount, then estimate biases as functions of this plug-in quantity. Thus, we actually estimate a warped version of the model, as illustrated in the right panel of Figure 1. Essentially, we center around the mean observed fluorescence intensities of each comparison group (the straight line in the right panel of Figure 1) and treat deviations from this mean as bias. The distinction between the original and warped versions of the model is subtle, and we show that inference performed on data normalized with our model will correctly identify the presence and direction of differential expression; this is a crucial property not guaranteed by MA-based methods [11]. Also, as seen in simulations below, this is achieved without under- or overfitting, as can happen with classic ANOVA-based normalization methods.

A consequence of this work is the statistical justification for a more efficient dye-swap design. Specifically, we show that dye bias can be removed without technical replication of sample pairs as is required in a traditional dye-swap experiment, as long as there is a simple balance in the experimental design. One example has half of the arrays under one dye configuration and the other half under the swapped configuration. In [11], we proposed the Common Array Dye Swap (CADS), an analogous method for a direct comparison experiment utilizing the usual dye-swap design by technical replicates. Since the present method targets an 'efficient dye-swap design', we call it the efficient Common Array Dye Swap (eCADS). eCADS is also an extension of CADS to general experimental designs.

Functional ANOVA (fANOVA) models are not new, and extensive results have been provided as to their features [12]. Our major contribution is the formulation of the two-channel microarray experiment in terms of the fANOVA model and the consideration of estimation in the absence of its functional arguments.

## Results and discussion

### Simulated example

We begin by applying the proposed eCADS normalization method to a simulated example, generated according to the eCADS model (equation 2). The model requires RNA amounts *x*_*il *_for each gene *i *and comparison group *l*, as well as dye- and array-specific functions that translate RNA amount into fluorescence intensity. In each of 100 simulations, 14 arrays were formed making direct comparisons of two groups. There are seven arrays under one dye configuration (with group 1 labeled red and group 2 labeled green) and seven arrays under the reverse dye configuration. This balanced (in dye configuration) aspect of the design is key to the operating characteristics of eCADS that we present below. This is an example of an 'efficient dye-swap', in that no technical replicates were used (see the section 'The efficient Common Array Dye Swap').

**Figure 2:**
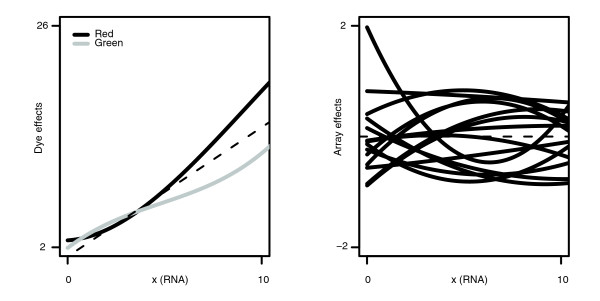
Functions used in simulation. Functions used in simulated example for dye (left) and array (right) effects. The actual dye 'effect' functions (the *δ*s in equation 2) are the dye-specific deviations from the average curve. The array functions sum to zero at any point on the *x*-axis.

RNA amounts *x*_*i*2_, *i *= 1,2,...,5000, for group 2 were randomly generated uniformly between 0 and 10; as described in the 'Model formulation' section below, these are merely conceptual quantities. Of the 5,000 genes, 70% were randomly selected to be null, with *x*_*i*1 _= *x*_*i*2_. For the remaining 30% of differentially expressed genes, RNA amounts for group 1 were set equal to RNA amounts for group 2, plus random deviates uniformly distributed between -1/2 and 5. The dye and array functions used in one of the simulations are shown in Figure 2. The same dye functions and mean values were used for each of the simulations, while the array functions were randomly selected in each simulation.

Note that there is asymmetric differential expression in this example. One of the assumptions behind MA methods is symmetric differential expression [11]. In this example, then, MA methods will artificially reshape the data. Meanwhile, a classic ANOVA-based normalization model [8,9,13] (see equation 1) will underfit the dye functions, since only a constant shift is allowed, and overfit the array functions, since no intensity-dependent relationships are acknowledged (see the section 'Model formulation' for details).

**Figure 3:**
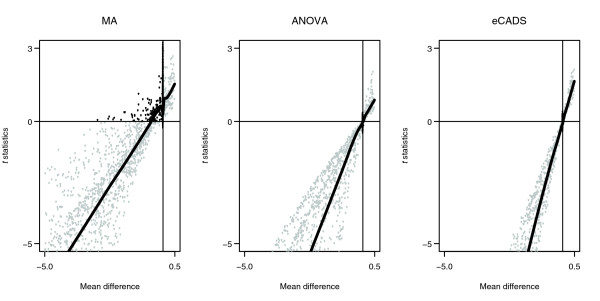
Summary of simulated *t*-statistics. Comparison of *t*-statistics (averaged across 100 simulations) and true mean differences after MA (left), ANOVA (middle), and eCADS (right) normalization. Black points represent genes for which the sign of differential expression has not been preserved. Plots for MA show systematic shift, indicating bias.

We normalized the data using each of MA smoothing, ANOVA, and eCADS. Figure 3 compares the average *t*-statistics across simulations to true RNA differences after each normalization method. Since *t*-statistics are just mean difference estimates divided by standard errors, we expect the *t*-statistics to share the same sign as the true mean differences; we see a scatter instead of a straight line because of the random variability in the simulation. Black dots indicate genes for which the sign of differential expression relationships was not preserved. There is a systematic deviation away from the origin in the figure for MA smoothing, indicating that this normalization method results in biased inference. No substantial bias is apparent after normalizing with the ANOVA model. It is clear from this figure that eCADS is unbiased. We provide a general result below that states that inference after eCADS normalization will correctly identify the presence and direction of differential expression.

**Figure 4:**
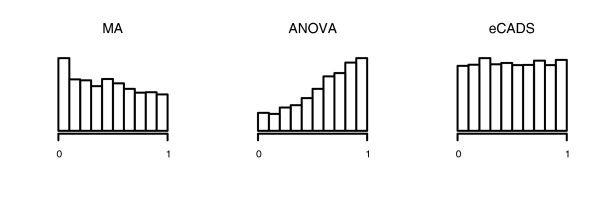
Summary of simulated null histograms. Histograms of null *p*-values after MA (left), ANOVA (middle), and eCADS (right) normalization in one simulation. Neither the MA nor ANOVA null *p*-values are uniformly distributed (KS significance approximately zero), while eCADS null *p*-values are (KS *p *= 0.86).

Figure 4 shows representative (from a single simulation) histograms of *p*-values for the null genes, after normalization by the three methods. One check on the validity of a normalization method is its effect on null *p*-values. In particular, a normalization method should preserve the uniform distribution of null *p*-values [14]. The null *p*-values in this simulation after MA normalization are not uniformly distributed, with Kolmogorov-Smirnoff (KS) significance approximately zero. The histogram shape suggests that signal has been artificially created in the null genes. The ANOVA model likewise does not produce uniformly distributed null *p*-values, with KS significance again approximately zero. The histogram shape suggests overfitting. eCADS, on the other hand, preserves the uniformity of the null *p*-values (KS *p *= 0.86). These results persisted across simulations. The KS test for uniformity of null *p*-values rejected its null hypothesis at the 5% level in 100, 100, and 8 simulations for the MA, ANOVA, and eCADS methods, respectively, where 5 rejections were expected by chance. We note also potential issues with dependence across genes that could arise due either to ANOVA approaches modeling array effects as factors instead of functions or to MA methods confounding biological effects with array effects.

#### Minor imbalances do not create bias

As detailed below, eCADS preserves differential expression relationships under a certain kind of balance in experimental design. The simulated examples satisfy this balance, with half of the arrays under one dye configuration and half under the reversed configuration. In certain situations, it may not be possible to have exact balance; for example, there may be an odd number of samples available. To investigate the effect of minor imbalances, we repeated the above simulation for the case where there are seven arrays in one configuration and six in the other, with all other parameters held constant. No additional bias was apparent, as the plot corresponding to that in Figure 3 was qualitatively indistinguishable from Figure 3. Also, the KS tests indicated null *p*-values for eCADS. This serves as informal support for eCADS in situations where perfect balance is not possible.

### Prostate development example

As a real example, we illustrate eCADS on a study of prostate development in mice. The data were kindly provided by the Peter S Nelson laboratory at the Fred Hutchinson Cancer Research Center (data available in Additional Data File 2). Six two-channel microarrays were formed, each comparing the prostate of a separate thirty-day-old mouse to fourteen-day post-conception embryonic controls; thus, twelve mice in total were involved. Three of the arrays labeled thirty-day samples red and controls green, and three arrays labeled 30-day samples green and controls red, making this an 'efficient dye-swap' experiment.

**Figure 5:**
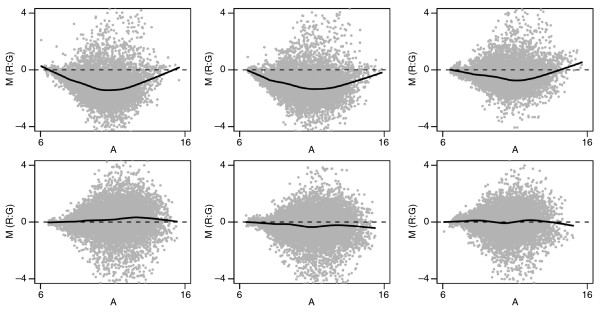
MA plots for mouse prostate development study. The three arrays from one dye configuration are in the top row, while those from the reversed configuration are in the second row. There is apparently asymmetric, intensity-dependent differential expression in this example.

Figure 5 shows MA plots for the raw data. The three arrays from one dye configuration are shown in the top row, and the three arrays from the reversed configuration are in the bottom row. There is a pronounced nonlinear trend in the first configuration (top row). If this were due exclusively to dye bias, then this trend would be reversed when the dyes were swapped. This would result in a nonlinear trend in the second configuration (bottom row) that is a symmetric reflection of the configuration-one trend about the horizontal zero line. This is clearly not the case here. Hence, the persistent asymmetry in Figure 5 suggests asymmetric and intensity-dependent differential expression. MA methods will treat the asymmetry as bias [11]. Assuming the asymmetry is 'real', MA methods will, therefore, artificially reshape the data, causing large-scale erroneous modifications of the biological signal.

**Figure 6:**
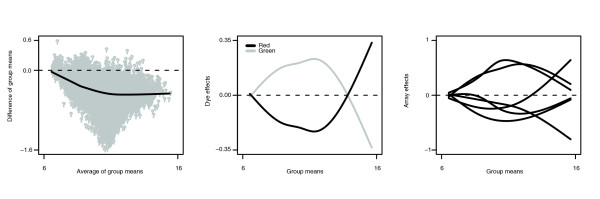
Estimated group means and bias functions for mouse data. The left panel is an MA plot comparing the 'warped RNA' (group means adjusted gene-by-gene for bias) for the two comparison groups. The middle panel shows the estimated dye effect functions, and the right panel shows the estimated array effect functions.

Figure 6 summarizes the results of applying eCADS to these data. The left panel is an MA plot comparing the estimated warped RNA amounts, equivalent to means within the two comparison groups after normalization. As suggested above, the model identifies substantial asymmetric differential expression, with the bulk of the scatterplot centered below zero; this corresponds to underexpression in 30-day mice relative to 14-day embryos. Widespread differences in expression would not be unexpected when comparing such disparate stages of development. The estimated dye functions are shown in the middle panel and the estimated array functions in the right panel. In terms of Figure 1, the dye functions are the *δ*s. They thus represent deviation from the group means due to dye and sum to zero at any point on the *x*-axis. Note that the asymmetry is taken into account through the group means themselves, corresponding to the 'average dye function' *d *in Figure 1. The array functions are the *a *in Figure 1 and also sum to zero at any point on the *x*-axis.

### Microarray Quality Control project example

Our second example comes from the Microarray Quality Control (MAQC) project [15]. The MAQC project compared two RNA samples, a total human reference and a human brain reference. We obtained data for direct comparisons on 30 Agilent two-color microarrays, where 10 arrays each were processed at 3 different sites. At each site, five arrays were formed in each dye configuration. We used eCADS with model terms for each of dye, site, and array to analyze 7,622 genes after filtering for single-probe, unreplicated genes with no quality issues.

**Figure 7:**
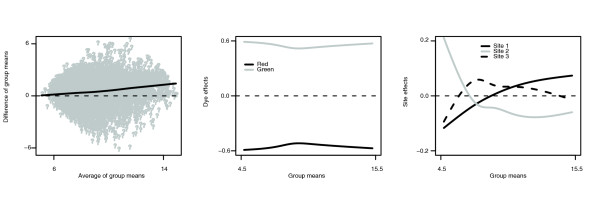
Estimated group means and bias functions for MAQC data. The left panel is an MA plot comparing the 'warped RNA' (group means adjusted gene-by-gene for bias) for the two comparison groups. The middle panel shows the estimated dye effect functions, and the right panel shows the estimated site effect functions.

**Figure 8:**
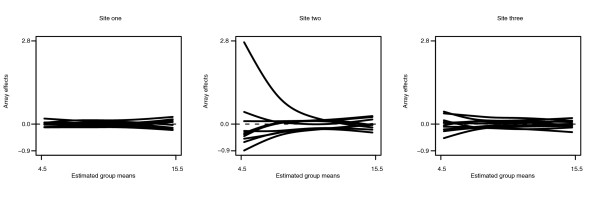
Estimated array functions for MAQC data. The estimated array effect functions by site. Note that site two has substantially more array-to-array variability than the other two sites.

Figures 7 and 8 are analogous to Figure 6 for the prostate data. It can be seen that the intensity-dependent trends, including intensity-dependent differential expression, hold here as well. Therefore, the assumptions of the ANOVA and MA approaches again appear to be violated in this example. Meanwhile, eCADS appears to capture intensity-dependent trends with no obvious numerical issues.

The examples that we have considered are similar in that they exhibit nontrivial levels of differential expression. The advantages of eCADS over existing methods are especially notable in such situations, since it is able to distinguish between biological signal and bias. Applying MA methods will artificially reshape all relationships, impacting the validity of any downstream analyses. We emphasize that eCADS is not restricted to experiments with nontrivial levels of differential expression but is generally applicable to any valid design. Even in examples where the MA assumptions hold, it is possible that eCADS will yield greater sensitivity and specificity through its ability to model the different sources of bias. eCADS will also maintain an advantage over gene-by-gene ANOVA methods by directly estimating the relevant bias functions instead of using factor terms.

## Conclusion

We have developed eCADS as a flexible and generally applicable approach to normalizing two-channel microarrays. In contrast with popular existing normalization methods motivated mostly by pictures and intuition, we have provided an approach based on an explicitly defined model, making it easier to state assumptions behind the method and its operating characteristics. In contrast with existing model-driven normalization methods, eCADS naturally incorporates intensity-dependent relationships between genes without overfitting.

eCADS can be applied to any valid experimental design, including direct comparisons, indirect comparisons, comparisons of more than two groups, and comparisons taking into account other relevant variables. Furthermore, we show below that, under a simple kind of balanced design, eCADS removes biases in such a way that differential expression relationships are preserved. This is achieved without the need for technical dye-swaps and without restrictive assumptions. Software implementing eCADS is available from the authors' website[16].

The ideas employed here have potential application in several other related settings. For example, single-channel microarrays have their own associated biases [17], but similar intensity-dependent relationships would be expected to hold. Typically, each array is normalized to a baseline array [18], or all arrays are normalized to each other in an iterative manner [19], but experimental design is not commonly used directly in single-channel microarray normalization methods; a recent exception is [20]. Similarly, Agilent's two-channel array CGH product uses rank-invariant features to smooth MA plots [21]. Also, the scatterplot smoothing routine of [19] was recently applied in the context of 'ChIP-chip' experiments using Affymetrix tiling arrays [22]. Protein mass-spectrometry [23] is yet another example where, as with microarrays, we expect a functional relationship between the biological quantity of interest (here, peptide abundance) and the observed outcome (peak intensity). Intensity-dependent biases are commonly seen, and scatterplot smoothing approaches have been considered [24].

## Materials and methods

### Model formulation

A two-channel microarray experiment produces data representing true expression levels that have been translated and distorted by various technological processes. In particular, we do not observe the true total (or even relative) counts of RNA molecules. We observe some function of these quantities in the form of a fluorescence intensity, where the function we observe incorporates various technical aspects of the experiment, including systematic biases. A minimal requirement is that microarray data preserve relative expression relationships. In particular, we want to be able to detect the presence and direction of true differential expression. This can be formalized by the requirement that the sign of relative relationships be preserved.

Our proposed statistical model for the observed expression values from a microarray experiment can be motivated by considering the early ANOVA models proposed for microarrays [8,13]. Let *y*_*ijkl *_be the observed log expression measurement, a single-channel fluorescence intensity, for gene *i *on array *j *labeled with dye *k *in comparison group *l*. We begin with the model:

(1) *y*_*ijkl *_= *μ*_*i *_+ *d*_*k *_+ *t*_*il *_+ *a*_*ij *_+ *ε*_*ijkl*_,

*i *= 1,2,...,*m*, *j *= 1,2,...,*n*, *k *= 1,2, *l *= 1,2. For simplicity, let *k *= 1 indicate red dye (*k *= 2 green). This is the ANOVA model of [8], with some of the components collapsed together. Thus, the *μ*_*i *_represent gene-specific baseline means, *d*_*k *_the dye effects, *t*_*il *_the group effects (a group main effect together with its gene × group interaction), *a*_*il *_'spot' effects (an array main effect together with its gene × array interaction), and the *ε*_*ijkl *_random error.

The spot effects *a*_*ij *_are allowed to change for every gene-array combination. However, there is evidence that such effects are functionally related within each array [2]. As such, fitting every point individually will result in an overfit. On the other hand, the dye effects *d*_*k *_are only allowed two values, one for each dye. There is also evidence for intensity-dependent dye effects [2-4]. As such, restricting dye changes to constant shifts will result in an underfit. A more flexible dye term could be used instead in equation 1. However, together with the overfit array terms, this would quickly use up all available degrees of freedom for model fitting.

A natural way to incorporate intensity-dependent relationships is to replace the factor terms in equation 1 with functions of the underlying RNA amounts, creating the fANOVA model:

(2) *y*_*ijkl *_= *d*(*x*_*il*_) + *δ*_*k*_(*x*_*il*_) + *a*_*j*_(*x*_*il*_) + *ε*_*ijkl*_,

where *x*_*il *_is the average RNA amount for gene *i *in group *l*. In terms of the model given in equation 1, *μ*_*i *_+ *t*_*il *_is now represented by *d*(*x*_*il*_), *d*_*k *_by *δ*_*k*_(*x*_*il*_), and *a*_*ij *_by *a*_*j*_(*x*_*il*_). General intensity-dependent biases due to differences between the dyes can now be flexibly modeled with the *δ*_*k*_(*x*_*il*_). Similarly, array-specific biases that are intensity-dependent can be modeled using the *a*_*j*_(*x*_*il*_), without the use of separate factor terms for every gene-array combination. To make model of equation 2 identifiable, we require that

$$\sum_{k=1}^{2}\delta_{k}(x)=0$$

and

$$\sum_{j=1}^{n}a_{j}(x)=0$$

for any argument *x*.

Note that 'RNA amount' *x*_*il *_is an abstract quantity meant only to help conceptualize the problem. Ideally, 'RNA amount' would mean the average number of RNA molecules available in group *l *for hybridization to the spot that characterizes gene *i*. Practically, we require only an 'RNA-equivalent amount', a bias-free monotone function of RNA count. As will be detailed below, we actually estimate a translated version of the model in equation 2 that is in terms of RNA-equivalent arguments that can be estimated from the data. Having said that, for convenience, we refer to the *x*_*il *_as 'RNA amount' throughout the paper.

While the form of the model in equation 2 is simple enough, a major problem is immediately apparent: we do not know the RNA amounts *x*_*il*_. If we did know the *x*_*il*_, we would preprocess the single-channel data in such a way that dye and array biases are removed, but relationships between the comparison groups are not affected. In terms of the model in equation 2, this can be accomplished by subtracting off the terms *δ*_*k*_(*x*_*il*_)+*a*_*j*_(*x*_*il*_) (leaving us with the quantities of interest, *d*(*x*_*il*_)). This would remove bias from both the individual observations and the average differences between comparison groups.

Note that MA-smoothing methods make assumptions about the form of the *x*_*il*_. For example, MA-smoothing methods assume that the gene-specific group differences *x*_*il*_-*x*_*il' *_are symmetric about zero and that these differences do not change systematically with gene-specific group averages (*x*_*il*_+*x*_*il'*_)/2 [11]. Since the *x*_*il *_are unknown, and no information about them is available without performing a dye-swap, the MA-smoothing assumptions are unverifiable in practice. Our estimation procedure does not assume any particular structure for the *x*_*il*_. The *x*_*il *_are merely arguments for the functions to be estimated. Our method thus applies regardless of whether there is any asymmetry, intensity-dependent differential expression, unequal variability between groups, more than 50% differential expression, and so on.

#### The efficient Common Array Dye Swap

In what can generally be called a 'dye swap', dye configuration is swapped in some arrays relative to others. That is, on some arrays, the red dye is used for group 1 and the green dye for group 2, while for other arrays this configuration is reversed. Typically, a 'technical dye-swap' is carried out, where the swapping occurs on technical replicate arrays. The general model in equation 2 is valid as long as there are arrays under both dye configurations. In particular, no technical replication is necessary, obviating the need for a technical dye-swap design. One simple implementation has half the arrays under one dye configuration (group 1 labeled red and group 2 green, say, in a direct-comparison experiment) and the other half under the other configuration. We define an 'efficient dye-swap' as swapping dye configuration on arrays that represent biological, rather than technical, replicates.

In [11], we proposed the CADS method for normalization when technical dye-swaps are used in direct-comparison experiments (with both comparison groups on the same array). The CADS model also uses functions of RNA amount to represent intensity-dependent biases. However, CADS is restricted to direct comparisons with technical dye-swaps and does not easily incorporate additional covariates or sources of bias. Since the present method is targeted at efficient dye-swap designs, we refer to it as eCADS; our use of the term 'efficient' is intended as a distinction between 'efficient'and 'technical' dye-swaps, not as a claim of efficiency of an estimator as used in statistics. eCADS is still applicable to direct-comparisons and technical replicates and, thus, can be seen as a generalization of CADS. As we discuss below, the eCADS model is estimated in a two-stage process. Under a simple form of balanced design, this estimation procedure preserves differential expression relationships in expectation.

eCADS can be applied to any valid experimental design using two-channel microarrays. Examples include: direct comparison experiments in which two groups are compared on the same array; indirect comparisons using a reference design (the dye used for the reference group must be swapped; and experiments in which there are more than two comparison groups. Additional covariates or sources of bias are also naturally incorporated. Furthermore, any feature of the model can be represented as either an intensity-dependent function or a traditional factor term.

We have shown in previous work that a technical dye-swap is necessary for removing dye bias in general from a single pair of samples [11]. With eCADS, we provide a more general result. Namely, dye bias can be removed from a set of sample pairs with dye-swaps on biological replicates. Technical dye-swaps are frequently avoided due to the inconvenience of making technical replicate arrays. MA methods are often used instead, at least partly due to their requiring only one array per sample pair. However, we have shown that MA methods require strong assumptions that cannot be validated in practice. eCADS handles the same intensity-dependent biases that are targeted by MA methods and also requires only one array per sample pair. In addition, eCADS does not require the restrictive assumptions of MA methods.

### Estimation

#### Unknown functional arguments

It is usually the case in the regression setting that one wants to characterize the association between the dependent and independent variables. In our setting, fluorescence intensity is the dependent variable, and RNA amount is the independent variable; recall comments about our use of 'RNA amount' in the section 'Model formulation'. However, for purposes of normalization, our goal is to estimate the model terms representing bias and remove them. As it turns out, we are able to estimate and remove bias terms from the eCADS model without having to directly observe or estimate the independent variable. This is done by estimating a translated version of the independent variable, borrowing strength across arrays, and redefining the model to be in terms of the translated version.

The left panel of Figure 1 illustrates a hypothetical realization of the model in equation 2. On the *x*-axis is RNA amount, and each of the component functions of the model in equation 2 for a single array are labeled. The fluorescence intensities *y*_*R *_(red) and *y*_*G *_(green) are the sum of the average dye function *d*, the dye-specific functions *δ*_*R *_or *δ*_*G*_, and the array-specific function *a*. The fluorescence intensity for a particular gene labeled red, say, is the evaluation of *y*_*R *_at that gene's RNA amount, plus random error.

While we cannot estimate the actual RNA amounts for each gene, we can translate the model in equation 2 so that it is in terms of a quantity that we can estimate. Specifically, we redefine the model in equation 2 to be defined in terms of *d*(*x*_*il*_) instead of *x*_*il*_. As illustrated in the right panel of Figure 1, this transformation of the model warps the *x*-axis in the left panel of Figure 1, leaving the fluorescence intensities unchanged. That is, the curves from the left panel have been reshaped by changing the positions of their arguments on the *x*-axis. Since we have reshaped around *d*(*x*_*il*_), the new average dye function is the line of equality. Also, we have not affected the qualitative characteristics of the dye- and array-specific functions. Having translated the model, we proceed with the two-stage estimation process: first, estimate the warped RNA amounts; and second, estimate the translated version of the model in equation 2, plugging in the warped RNA estimates.

In the simplest case, with group *l *labeled with both dyes on an equal number of arrays, we can estimate *d*(*x*_*il*_) by simply averaging all observations for gene *i *in group *l*; the dye and array effects cancel out. More generally, we estimate the *d*(*x*_*il*_) by fitting separate regression models to each gene (see the appendix in Additional data file 1 for details). As a result, the 'warped RNA amounts' can be thought of as simple group means, adjusted gene-by-gene for bias. Thus, in general, eCADS begins with the fitted values from gene-specific regressions, analogous to the ANOVA approach. However, these gene-specific estimates are smoothed across genes by plugging them into the functions of the eCADS model.

#### Parameterizing the model

Regression or ANOVA models are typically represented by model matrices. The same principles apply in the microarray setting [25]. An efficient dye-swap design can be characterized by a standard design matrix *Z *with rows for each array channel and columns for the comparison groups, dyes, and arrays. Specifically, with *n *arrays and *p *comparison groups, define *Z *to be the 2*n *× (*n *+ *p *+ 2) matrix with *h*th row equal to:

$$[z_{g_{1}h}...z_{g_{p}h}z_{d_{1}h}z_{d_{2}h}z_{a_{1}h}...z_{a_{n}h}],$$

*h *= 1,2,...,2*n*. The component $z_{g_{l}h}$ indicates whether the *h*th channel is from comparison group *l*, $z_{d_{k}h}$ indicates whether the *k*th dye was used for labeling the *h*th channel, and $z_{a_{j}h}$ indicates whether channel *h *is from array *j*. As an example, an experiment making direct comparisons between two groups using two arrays would have:

$$Z=\left(\begin{array}{cccccc} 0 & 1 & 0 & 1 & 1 & 0\\ 1 & 0 & 1 & 0 & 1 & 0\\ 0 & 1 & 1 & 0 & 0 & 1\\ 1 & 0 & 0 & 1 & 0 & 1 \end{array}\right).$$

We express the functions of the model of equation 2 in terms of basis matrices. By combining these basis matrices with the information in the model matrix *Z*, we can write the eCADS model as a typical regression model in matrix form. Estimation is carried out by minimizing a sum-of-squares criterion subject to identifiability constraints on the regression parameters (details are given in the appendix in Additional data file 1). We emphasize that the eCADS model can be applied to any 'valid' (derived from an experimental design that allows estimation of the parameters of interest) model matrix *Z*. This includes direct comparisons, indirect comparisons (reference designs), and multiple comparison groups.

The fANOVA model and its basis matrix representation allow for flexible choices of the form of its component functions, as well as for the inclusion of additional sources of bias. The dye functions are defined as being monotone, and this could be explicitly incorporated into the model, as discussed in chapter 6 of [26]. Time-dependent biases, as can arise when forming arrays over periods of weeks or months, could be incorporated by adding functions of time. Spatial biases could be incorporated through the inclusion of a two-dimensional smoother, as in [27]. In the analyses that follow, we use natural cubic spline bases [28] with five degrees of freedom.

### Operating characteristics

#### eCADS preserves differential expression relationships

We now consider making inference on data normalized with eCADS. Because fluorescence intensities are nonlinear warped versions of RNA amount even with perfect normalization, it is not possible to preserve relative fold-change amounts in terms of direct RNA counts. It is possible, however, to preserve the sign and/or existence of differential expression. Specifically, after eCADS normalization, the expected value of a test statistic for a particular gene that compares two groups should equal the sign of the true difference in RNA amount. This means that, in expectation, null genes are called null, overexpressed genes are called overexpressed, and underexpressed genes are called underexpressed. Since differential expression methods are almost always based on sample averages [29], we assume that the test statistics are based on sample averages.

It can be shown (see the appendix in Additional data file 1) that eCADS normalization preserves the sign of differential expression in expectation under a simple kind of balance in experimental design that we refer to as 'balance with respect to comparison group'. An efficient dye-swap design is 'balanced with respect to comparison group' if each experimental level for any one factor is repeated the same number of times for each comparison group. For example, a direct comparison of two groups with *n *arrays that follows the model in equation 2 is balanced with respect to comparison group if *n*/2 arrays have one dye configuration, and *n*/2 have the other. If indirect comparisons are made using reference designs, dye configuration must still be swapped. Suppose, for example, that there are three comparison groups, with *n*_1 _arrays for group 1, *n*_2 _arrays for group 2, *n*_3 _and arrays for group 3, with the same reference sample used for all arrays. Balance with respect to comparison group requires that *n*_1_/2 of the arrays corresponding to group 1 have the group 1 target labeled red, while the other *n*_1_/2 have reference labeled red, for example. The arrays in the other groups must similarly be broken into equal groups by dye configuration.

To further illustrate the generality of the eCADS model, consider the following example. In making direct comparisons between two groups, suppose that there is bias due to dye, array, and array batch, with *B *batches. Suppose also that we would like to adjust for gender when comparing the groups. We thus consider the model:

(3) *y*_*ijklrs *_= *d*(*x*_*il*_) + *δ*_*k*_(*x*_*il*_) + *a*_*j*_(*x*_*il*_) + *c*_*r*_(*x*_*il*_) + *b*_*s*_(*x*_*il*_) + *ε*_*ijklrs*_,

where *c*_*r *_corresponds to the *r*th gender, *r *= 1,2, and *b*_*S *_corresponds to the *s*th array batch, *s *= 1,2,...,*B*. As with the other model terms, $\underset{r}{\Sigma }c_{r}(x)=\underset{s}{\Sigma }b_{s}(x)=0$ for any argument *x*. Normalization by eCADS would remove the biases due to dye, array, and batch. Inference would then be carried out by a weighted average of group differences within each gender, as with a gene-specific linear regression. Balance with respect to comparison group here requires: *n*/2 arrays in each dye configuration; *n*/*B *arrays in each array batch; and *n*/2 males in group 1, *n*/2 females in group 1, *n*/2 males in group 2, and *n*/2 females in group 2. Note that it does not matter how these factors are arranged with respect to one another.

The question remains of the penalty incurred when perfect balance with respect to comparison group is not possible due, for example, to an odd number of arrays. In general, using plug-in estimates for RNA amounts *x *is analogous to regressing on a covariate that has been measured with error. Outside of very simple settings, it is not possible to fully characterize the bias that results from such measurement error problems. However, as seen in the simulations above, there does not appear to be much of a penalty for having minor imbalances.

## Additional data files

The following additional data are available with the online version of this paper. Additional data file 1 is an appendix, containing mathematical details of the eCADS method. Additional data file 2 contains the prostate development data in comma-delimited text form. Additional data file 3 describes the prostate data format.

## Supplementary Material

Additional data file 1 Mathematical details of the eCADS method Click here for file

Additional data file 2 The data are in comma-delimited text form Click here for file

Additional data file 3 Description of the prostate data format Click here for file
