# Supplementary material for: Normalization of two-channel microarrays accounting for experimental design and intensity-dependent relationships
Source: Genome Biol. 2007 Mar 28;8(3):R44. doi: 10.1186/gb-2007-8-3-r44 (PMC1868928; doi:10.1186/gb-2007-8-3-r44)
Supplement: Additional data file 1 — Mathematical details of the eCADS method [file gb-2007-8-3-r44-S1.pdf]

Appendix: Normalization of Two-Channel Microarrays Accounting for Experimental Design and Intensity-Dependent Relationships

Alan R. Dabney
Department of Statistics
Texas A&M University, College Station, TX 77843
adabney@stat.tamu.edu

John D. Storey
Department of Biostatistics
Department of Genome Sciences
University of Washington, Seattle, WA 98195
jstorey@u.washington.edu

Translating the eCADS model

While we do not know the x_{il}, we are able to estimate monotone-increasing functions of them, as follows. Let $x^*_{il} = d(x_{il}) = (d(x_{il}) + \delta_1(x_{il}) + d(x_{il}) + \delta_2(x_{il}))/2$. Now define functions d^*, δ^*_k, and a^*

$$d^*(x^*_{il}) = x^*_{il} = d(x_{il})$$
$$\delta^*_k(x^*_{il}) = \delta_k(x_{il})$$
$$a^*_j(x^*_{il}) = a_j(x_{il}).$$

The new dye functions $d^*(x^*_{il}) + \delta^*_k(x^*_{il})$ are also monotone-increasing, and preserving this monotonicity is more important than estimating the true dye functions. These definitions also preserve the constraints $\sum_{k=1}^{2} \delta^*_k(x) = 0$ and $\sum_{j=1}^{n} a^*_j(x) = 0$ for any argument x. We can therefore translate

the model in equation ((2)) from the main document into

$$\begin{aligned} y_{ijkl} &= d^*(x^*_{il}) + \delta^*_k(x^*_{il}) + a^*_j(x^*_{il}) + \epsilon_{ijkl} \\ &= x^*_{il} + \delta^*_k(x^*_{il}) + a^*_j(x^*_{il}) + \epsilon_{ijkl}, \end{aligned} \tag{a}$$

where the second equality follows by the definition of d^*. We proceed with the two-stage approach: (i) estimate the x^*_{il}, (ii) fit model (a) using the $\hat{x}^*_{il}$ as plug-in estimates of the x^*_{il}.

Estimating the warped RNA amounts

In general, to estimate the x^*_{il}, we use fitted values from gene-specific ANOVA or regression models. One simple model choice is based on ANOVA model in equation ((1)) of the main document:

$$y_{ijkl} = \mu_i + d_k + t_{il} + \epsilon_{ijkl}. \tag{b}$$

The spot effects a_{ij} are not necessary for our purposes here and are excluded from the model. Additional covariates or sources of bias could be added to the gene-specific model as necessary. Recall that $x^*_{il} = d(x_{il})$ in the eCADS model corresponds to $\mu_i + t_{il}$ in the ANOVA model. Hence, we estimate the x^*_{il} with the fitted values $\hat{\mu}_i + \hat{t}_{il}$ from model (b). The estimated "warped RNA amounts" can thus be thought of as group-specific means, adjusted gene-by-gene for bias.

We note that in our previous work on the CADS method (Dabney & Storey 2007), we defined all intensity-dependent functions to be in terms of *subject-specific* RNA amounts, x_{ijl}. Since CADS targets technical dye-swap experiments, where comparisons are made between paired arrays, it is natural to think of its arguments as being subject-specific. In the present setting, we could also use subject-specific RNA amounts. This requires some further assumptions. However, importantly, the choice between population- or subject-specific RNA amounts does not affect the targets of interest, $d(x_{il})$, and hence does not affect the operating characteristics stated here for eCADS. For details, see Dabney (2006).

Basis matrix representation of the fANOVA model

Suppose we can write the component functions of (a) in terms of basis matrices:

$$\begin{aligned} \delta^*_k(x^*_{il}) &= \boldsymbol{B_{\delta il}}\ \boldsymbol{\beta_{\delta_k}} \\ a^*_j(x^*_{il}) &= \boldsymbol{B_{ail}}\ \boldsymbol{\beta_{a_j}}. \end{aligned}$$

Here, for example, $\boldsymbol{B_{\delta il}} = [b_{\delta 1}(x^*_{il})\ b_{\delta 2}(x^*_{il})\ \ldots\ b_{\delta q_\delta}(x^*_{il})]$ is the q_δ-dimensional basis component (a q_δ-dimensional row-vector) for the dye functions at the ith gene, evaluated at RNA amounts specific to group l. We can similarly define $\boldsymbol{\delta^*_k(x^*_l)}$ to be the vector-valued function with ith component equal to $\delta^*_k(x^*_{il})$, *etc.* This allows us to write

$$\boldsymbol{\delta^*_k(x^*_l)} = \boldsymbol{B_{\delta l}}\ \boldsymbol{\beta_{\delta_k}}$$
$$\boldsymbol{a^*_j(x^*_l)} = \boldsymbol{B_{al}}\ \boldsymbol{\beta_{a_j}},$$

where, for example, $\boldsymbol{B_{\delta l}} = [\boldsymbol{B^T_{\delta 1l}}\ \boldsymbol{B^T_{\delta 2l}}\ \ldots\ \boldsymbol{B^T_{\delta ml}}]^T$ is now a $m \times q_\delta$ matrix.

Now group the y_{ijkl} into single-channel chunks, $\boldsymbol{y_h}$, $h = 1, 2, \ldots, 2n$. Let $\boldsymbol{Z}$ be the model matrix describing the experiment, as defined in the section *Parameterizing the model.* Specifically, let $\boldsymbol{Z}$ be the $2n \times (n + p + 2)$ matrix with hth row equal to $\left[z_{g_1 h}\ \ldots\ z_{g_p h}\ z_{d_1 h}\ z_{d_2 h}\ z_{a_1 h}\ \ldots\ z_{a_n h}\right]$. Here, $z_{g_l h}$ is a scalar indicator of whether channel h comes from group l (1 for yes, 0 for no), $l = 1, 2, \ldots, p$. Similarly, $z_{d_1 h}$ and $z_{d_2 h}$ indicate whether channel h was labeled with the red or green dyes, respectively, and $z_{a_j h}$ indicates whether the hth channel profile comes from array j, $j = 1, 2, \ldots, n$. Define

$$\boldsymbol{G_{xh}} = \sum_{l=1}^{p} \boldsymbol{x^*_l}\ z_{g_l h}$$
$$\boldsymbol{G_{\delta_k h}} = \left[\sum_{l=1}^{p} \boldsymbol{B_{\delta l}}\ z_{g_l h}\right] z_{d_k h}$$
$$\boldsymbol{G_{a_j h}} = \left[\sum_{l=1}^{p} \boldsymbol{B_{al}}\ z_{g_l h}\right] z_{a_j h},$$

$k = 1, 2,\ j = 1, 2, \ldots, n,\ h = 1, 2, \ldots, 2n$. We can then rewrite (a) as

$$\begin{aligned}\boldsymbol{y_h} &= \boldsymbol{G_{xh}} + \sum_{k=1}^{2} \boldsymbol{G_{\delta_k h}} \boldsymbol{\beta_{\delta_k}} + \sum_{j=1}^{n} \boldsymbol{G_{a_j h}} \boldsymbol{\beta_{a_j}} + \boldsymbol{\epsilon_h} \\ &= \boldsymbol{G_{xh}} + \boldsymbol{H_h}\ \boldsymbol{\theta} + \boldsymbol{\epsilon_h},\end{aligned}$$

$h = 1, 2, \ldots, 2n$, where $\boldsymbol{H_h} = [\boldsymbol{G_{\delta_1 h}}\ \boldsymbol{G_{\delta_2 h}}\ \boldsymbol{G_{a_1 h}}\ \ldots\ \boldsymbol{G_{a_n h}}]$ and $\boldsymbol{\theta} = (\boldsymbol{\beta^T_{\delta_1}},\ \boldsymbol{\beta^T_{\delta_2}},\ \boldsymbol{\beta^T_{a_1}},\ \ldots,\ \boldsymbol{\beta^T_{a_n}})^T$. Finally, we can stack the $\boldsymbol{H_h}$ on top of each other to form $\boldsymbol{H} = [\boldsymbol{G_{\delta_1}}\ \boldsymbol{G_{\delta_2}}\ \boldsymbol{G_{a_1}}\ \ldots\ \boldsymbol{G_{a_n}}]$, where the $\boldsymbol{G}$ components are the corresponding stacked versions of the $\boldsymbol{G_h}$ defining $\boldsymbol{H_h}$. We similarly

define $\boldsymbol{G_x}$ as the stacked version of the $\boldsymbol{G_{xh}}$. This gives

$$\begin{aligned} \boldsymbol{y} &= \boldsymbol{G_x} + \sum_{k=1}^{2} \boldsymbol{G_{\delta_k}}\ \boldsymbol{\beta_{\delta_k}} + \sum_{j=1}^{n} \boldsymbol{G_{a_j}}\ \boldsymbol{\beta_{a_j}} + \boldsymbol{\epsilon} \\ &= \boldsymbol{G_x} + \boldsymbol{H}\ \boldsymbol{\theta} + \boldsymbol{\epsilon}. \end{aligned} \tag{c}$$

Least-squares solutions

Our estimation goal is to minimize the least-squares criterion

$$(\boldsymbol{y} - \boldsymbol{G_x} - \boldsymbol{H}\ \boldsymbol{\theta})^T(\boldsymbol{y} - \boldsymbol{G_x} - \boldsymbol{H}\ \boldsymbol{\theta})$$

with respect to $\boldsymbol{\theta}$ subject to the constraints $\sum_{k=1}^{2} \boldsymbol{\beta_{\delta_k}} = 0$ and $\sum_{j=1}^{n} \boldsymbol{\beta_{a_j}} = 0$. This can be done by replacing $\boldsymbol{\beta_{\delta_2}}$ with $-\boldsymbol{\beta_{\delta_1}}$ and $\boldsymbol{\beta_{a_n}}$ with $-\sum_{j=1}^{n-1} \boldsymbol{\beta_{a_j}}$ in equation (c), leading to

$$\boldsymbol{y} = \boldsymbol{G_x} + \boldsymbol{H_0}\ \boldsymbol{\theta_0} + \boldsymbol{\epsilon}.$$

Here, $\boldsymbol{H_0} = [\boldsymbol{G_{\delta_1}} - \boldsymbol{G_{\delta_2}}\ \boldsymbol{G_{a_1}} - \boldsymbol{G_{a_n}}\ \ldots\ \boldsymbol{G_{a_{n-1}}} - \boldsymbol{G_{a_n}}]$ and $\boldsymbol{\theta_0} = (\boldsymbol{\beta_{\delta_1}}^T, \boldsymbol{\beta_{a_1}}^T, \ldots, \boldsymbol{\beta_{a_{n-1}}}^T)^T$. Standard least-squares theory leads to the estimate

$$\hat{\boldsymbol{\theta}}_{\boldsymbol{0}} = (\boldsymbol{H_0}^T \boldsymbol{H_0})^{-1} \boldsymbol{H_0}^T (\boldsymbol{y} - \boldsymbol{G_x}).$$

eCADS preserves differential expression relationships

eCADS can be applied to any valid experimental design. For optimal results, however, the design should be balanced with respect to comparison group. When this balance holds, the gene-specific sample averages of the eCADS-normalized data equal the quantities of interest ($d(x_{il})$) in expectation. Thus, since $d(x_{il})$ is a monotone function of the true RNA amounts, the expected value of any test statistic based on sample averages that compares the expression level of a gene in different groups has the same sign as the true difference in RNA amount. This means that, in expectation, null genes will be called null, overexpressed genes called overexpressed, and underexpressed genes called underexpressed. Based on simulation work, minor imbalances do not affect this result. A detailed proof follows.

Let $\boldsymbol{Z}$ be the model matrix describing the experiment. Denote the hth row of $\boldsymbol{Z}$ by $\boldsymbol{z_h} = [\boldsymbol{z_{gh}}\ \boldsymbol{z_{dh}}\ \boldsymbol{z_{ah}}\ \boldsymbol{z_{ch}}\ \boldsymbol{z_{bh}}]$, $h = 1, 2, \ldots, 2n$. Here, $\boldsymbol{z_{gh}} = (z_{g_1h}\ z_{g_2h}\ \ldots\ z_{g_ph})^T$, $\boldsymbol{z_{dh}} = (z_{d_1h}\ z_{d_2h})^T$, and $\boldsymbol{z_{ah}} = (z_{a_1h}\ z_{a_2h}\ \ldots\ z_{a_nh})^T$. The component $\boldsymbol{z_{ch}}$ represents factors of interest, such as confounders, while the component $\boldsymbol{z_{bh}}$ represents biases additional to the dyes and arrays used. To be general,

suppose there are C additional factors of interest, where the rth factor of interest has p_{Cr} levels, $r = 1, 2, \ldots, C$. We associate with the uth level of the rth factor of interest the function c_{ru} and assume that $\sum_{u=1}^{p_{Cr}} c_{ru} = 0$, $r = 1, 2, \ldots, C$. Similarly, suppose there are B additional bias terms, where the sth bias factor has p_{Bs} levels, $s = 1, 2, \ldots, B$. We associate with the vth level of the sth additional bias factor the function b_{sv} and assume that $\sum_{v=1}^{p_{Bs}} b_{sv} = 0$, $s = 1, 2, \ldots, B$.

Let $\tilde{y}_{il}$ be the sample average of the eCADS-normalized data for gene i in comparison group l. Since we are assuming that test statistics will be based on sample averages, and since the factors of interest are balanced with respect to comparison group, it is sufficient to consider the test statistic $t_i = \tilde{y}_{il} - \tilde{y}_{il'}$ for comparing the expression level of gene i in groups l and l'. Let $n_{\delta_k l}$ be the number of times group l is labeled with dye k, and let $n_{a_j l}$ be an indicator of whether group l is on array j. Similarly, let $n_{c_{ru} l}$ be the number of times group l takes level u of the rth additional factor of interest. Finally, let $n_{b_{sv} l}$ be the number of times group l takes level v of the sth additional bias factor. We can write

$$\begin{aligned}\tilde{y}_{il} = x_{il}^* &+ \frac{1}{n}\sum_{k=1}^{2} n_{\delta_k l}(\delta_k^*(x_{il}^*) - \hat{\delta}_k^*(\hat{x}_{il}^*)) + \frac{1}{n}\sum_{j=1}^{n} n_{a_j l}(a_j^*(x_{il}^*) - \hat{a}_j^*(\hat{x}_{il}^*)) \\ &+ \frac{1}{n}\sum_{r=1}^{C}\sum_{u=1}^{p_{Cr}} n_{c_{ru} l}(c_{ru}^*(x_{il}^*) - \hat{c}_{ru}^*(\hat{x}_{il}^*)) + \frac{1}{n}\sum_{s=1}^{B}\sum_{v=1}^{p_{Bs}} n_{b_{sv} l}(b_{sv}^*(x_{il}^*) - \hat{b}_{sv}^*(\hat{x}_{il}^*)) + \bar{\epsilon}_{il}.\end{aligned}$$

If there is balance with respect to comparison group, then $n_{\delta_1 l} = n_{\delta_2 l}$, $n_{c_{11} l} = \ldots = n_{c_{1p_{C1}} l}$, …, $n_{c_{C1} l} = \ldots = n_{c_{Cp_{CC}} l}$, $n_{b_{11} l} = \ldots = n_{b_{1p_{B1}} l}$, …, and $n_{b_{B1} l} = \ldots = n_{b_{Bp_{BB}} l}$. If direct comparisons between two groups were made, then each group appears on every array, and $n_{a_1 l} = \ldots = n_{a_n l}$. For indirect comparisons or experiments with more than two groups, this will not be the case. However, in these cases, we can further assume that array functions sum to zero within each relevant subset of arrays. In practice, this assumption is not necessary, since array effects are expected to be minor in comparison to dye effects; any residual array effect will not change the monotonicity of d. Based on the sum-to-zero constraints on the model terms, we therefore have $\tilde{y}_{il} = x_{il}^* + \bar{\epsilon}_{il} = d(x_{il}) + \bar{\epsilon}_{il}$. A similar result holds for group l', so that $\mathrm{E}(T_i) = d(x_{il}) - d(x_{il'})$. The average dye function d is monotone increasing, and so

$$\mathrm{E}(T_i) \begin{cases} = 0 & \text{if} \quad x_{il} = x_{il'}, \\ > 0 & \text{if} \quad x_{il} > x_{il'}, \\ < 0 & \text{if} \quad x_{il} < x_{il'}. \end{cases}$$

References

Dabney, A. R. (2006). *The Normalization of Two-Channel Microarrays*, Ph.D. dissertation, University of Washington.

Dabney, A. R. & Storey, J. D. (2007). A new approach to intensity-dependent normalization of two-channel microarrays, *Biostatistics* **8**: 128–139.
