# Supplementary material for: Normalization of two-channel microarrays accounting for experimental design and intensity-dependent relationships
Source: Genome Biol. 2007 Mar 28;8(3):R44. doi: 10.1186/gb-2007-8-3-r44 (PMC1868928; doi:10.1186/gb-2007-8-3-r44)
Supplement: Additional data file 3 — Description of the prostate data format [file gb-2007-8-3-r44-S3.pdf]

Description of Prostate Development Data File

Additional data file 2 contains the prostate development data in comma-delimited text format. Since the data is currently unpublished, no gene indentification information is included. Instead, gene names are of the form "gene_1," "gene_2," *etc.* Sample names are given in the first row and are of the form "RT1," "GC4," *etc.* The first letter indicates dye color ("R" for red and "G" for green). The second letter indicates group membership ("T" for 30-day infant mice and "C" for control 14-day embryo mice). The third letter indicates array number, ranging from 1 to 6. The intensity values have been $\log_2$-transformed.
